# Gastrointestinal parasite infections and associated factors in fighting bulls over 7 years of monitoring in Southern Thailand

**DOI:** 10.14202/vetworld.2024.895-902

**Published:** 2024-04-25

**Authors:** Dhiravit Chantip, Nantaporn Chooruang, Kitikarn Sakuna, Warawut Sukmak, Wiruntita Bohman

**Affiliations:** 1 Department of Veterinary Clinical Sciences, Faculty of Veterinary Science, Rajamangala University of Technology, Srivijaya, Thungyai, Nakhon Si Thammmarat, Thailand; 2 Laboratory and Diagnostic Centre of the Teaching Animal Hospital, Faculty of Veterinary Science, Rajamangala University of Technology, Srivijaya, Thungyai, Nakhon Si Thammmarat, Thailand

**Keywords:** bullfighting, gastrointestinal parasites, geographical areas, prevalence, Thailand

## Abstract

**Background and Aim:**

Indigenous beef cattle engaged in bullfighting in Southern Thailand represent a distinctive and valuable breed. Gastrointestinal (GI) parasites, which are recognized as important pathogens, have a negative impact on the overall health and physical performance of these fighting bulls. This study aimed to estimate the prevalence of GI parasitic infections and identify factors associated with these infections in a fighting bull population in Southern Thailand.

**Materials and Methods:**

Fecal samples (n = 4,244) from fighting bulls were submitted to the Laboratory and Diagnostic Centre of the Teaching Animal Hospital, Faculty of Veterinary Science, Rajamangala University of Technology Srivijaya. We examined the samples using simple flotation and centrifugal sedimentation methods. Individual animal profiles and demographic data were collected.

**Results:**

The overall prevalence of GI parasitic infections was 93.2%. Nine GI parasites were identified as *Paramphistome* spp. [PP]. being the most prevalent (93.2%), and the highest annual prevalence occurred in 2019 (97.9%). The infection rates of various parasite species were significantly related to the years of study, geographic area, season, and age group (p < 0.05). The prevalence of parasitic infection was higher on the west coast (98.6%) than on the east coast (98.0%). PP, *Eurytrema* spp., *Strongyles* spp., and *Buxtonella* spp. infections differed significantly among the seven provinces of Southern Thailand (p < 0.05). The prevalence of GI parasitic infections was higher during the rainy season (98.5%) than during the summer (97.7%). Bulls aged 7.0–7.9 years and 8.0–8.9 years had the highest parasite infection rate (99.2%) compared with those aged 8.0–8.9.

**Conclusion:**

GI parasitic infections continue to be a significant health concern among fighting bulls in Southern Thailand. Regular epidemiological investigations are crucial for developing effective preventive programs and control strategies and providing basic knowledge for bull farmers.

## Introduction

Gastrointestinal (GI) parasites are the most common pathogens affecting cattle, leading to serious health problems such as malnutrition, anemia, reduced growth, and reduced reproductive performance in infected animals. Numerous studies have highlighted the high prevalence of GI parasite infections in tropical and subtropical countries, including Thailand [1–3]. Several key factors contribute to the spread of GI parasite infection in cattle, including altitude, geographical area, tropical climate conditions, age group, inappropriate population density, insufficient health practices, and a lack of knowledge among farmers [4–6].

In Southern Thailand, bullfighting is a unique traditional sport popular among both locals and tourists. The bulls selected for these fights are selected from male indigenous breeds known for good performance and fall within the age range of 4–6 years [7]. If the bull does not suffer frequent losses or premature death, it may continue to fight until the age of 14–15 years. Proper health care and disease prevention are essential for fighting bulls. Good health plays a key role in ensuring the optimal functioning of the circulatory system, facilitating the delivery of oxygen to nourish muscles and various parts of the body [8]. In turn, it enhances the bulls’ ability to engage in prolonged exercise and cope more effectively with the challenges encountered in competitive environments. A previous study reported the prevalence of GI parasite infections in fighting bulls in South Thailand [9]; however, there has been little long-term survey information on the associated factors. There is a critical need to deepen our understanding of the prevalence of GI parasite infections in fighting bulls in this region of Thailand and to address factors related to these infections. This knowledge is essential for the development of effective control and prevention strategies.

Therefore, the objective of the present study was to estimate the prevalence and investigate the factors associated with GI parasite infection in fighting bulls in southern Thailand.

## Materials and Methods

### Ethical approval

This study was approved by the Institutional Animal Care and Use Committee of the University of Technology Srivijaya, Thailand (Approval no. IAC). Samples were collected according to standard procedures to ensure the well-being of the cattle, with no harm inflicted during the process.

### Study period and location

**Figure-1:**
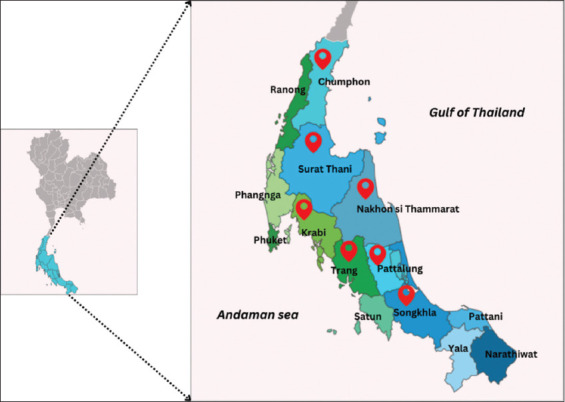
Map of southern Thailand highlighting the provincial locations of animal farms sampled in this study, with locations marked by red icons [Source: https://pixelmap.amcharts.com/].

The study was conducted from January 2016 to March 2023 in eight districts in Southern Thailand: Nakhon si Thammarat, Surat Thani, Phatthalung, Songkhla, Chumphon, Krabi, and Trang, as shown in Figure-1. The southern part of Thailand lies between latitudes of 5°–12° north and 98°–103° east. It is located between the Gulf of Thailand (East Coast) and the Andaman Sea (West Coast). The provinces of Nakhon Si Thammarat, Surat Thani, Phatthalung, Songkhla, and Chumphon are located on the east coast, while Krabi and Trang are located on the west coast. The climate in the southern part follows tropical monsoons, and the seasons vary between the two coastlines. On the west coast, there is a rainy season from April to November, and the summer lasts from December to March. On the east coast, there is a rainy season from May to December and the summer from January to April. The average temperature ranges from 25.6°C to 28.4°C, the average humidity ranges from 72.4% to 86.5%, and the average annual rainfall ranges from 2,197.0 to 2,707.0 mm.

### Sample size

Fecal samples from bulls were collected from eight provinces in Southern Thailand. The sample size was determined using the following equation:


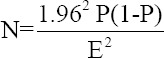


where N denotes the sample size, P denotes the expected prevalence, and E signifies the acceptable margin of error regarding prevalence within the fighting bull population. The sample size calculation was predicated on reported data indicating a 94.27% prevalence of GI parasite infection among fighting bulls in Southern Thailand [9], with a permissible error margin of 5%. As a result, the calculated minimum required sample size was 87 persons.

### Sample and data collection

Fresh fecal samples from 4244 fighting bulls, selected through simple random sampling were examined for GI parasites at the Laboratory and Diagnostic Centre, Faculty of Veterinary Science, Rajamangala University of Technology, Srivijaya. Laboratory technologists examined the samples for GI parasite eggs using simple flotation and sedimentation techniques. A simple floatation was performed using a saturated sodium chloride floatation solution, and GI parasite eggs were identified at 100× and 400× magnification using standard methods. Centrifugal sedimentation using tap water as the sediment solution was used for fluke egg detection. Age and other demographic information of the bulls were recorded.

### Statistical analysis

All data obtained during the study period were analyzed using IBM® Statistical Package for the Social Sciences® Statistics version 20 (IBM Corp., NY, USA) software. A non-parametric Chi-square test was applied to check the association between the variables (years of study, provincial and geographic location of the farms, season, and age group) and GI parasitic infection with a confidence level of 95%.

## Results

**Figure-2:**
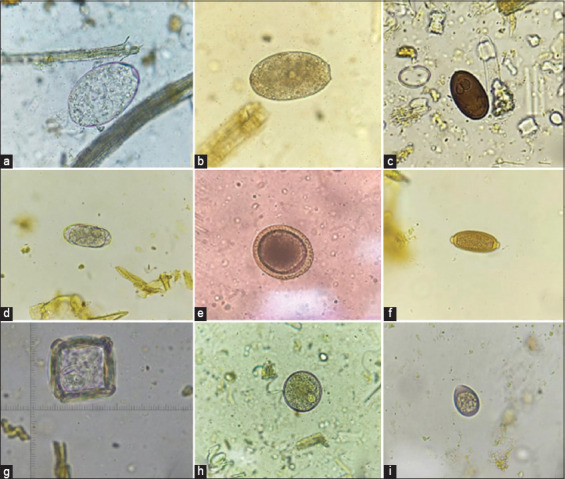
Gastrointestinal parasite eggs and oocytes were identified in samples from fighting bulls. The specific parasites detected include: (a) *Paramphistomum* spp., (b) *Fasciola* spp., (c) *Eurytrema* spp., (d) Strongyles, (e) *Toxocara* spp., (f) *Trichuris* spp., (g) *Moniezia* spp., (h) *Buxtonella* spp., and (i) *Eimeria* spp.

Nine GI parasites (*Paramphistome* spp. [PP], *Eurytrema* spp. [ET], *Fasciola* spp. [FL], Strongyles [SL], *Toxocara* spp., *Trichuris* spp. [TR], *Moniezia* spp. [MZ], *Buxtonella* spp. [BT], and *Eimeria* spp. [EM]) with an average prevalence of 19.6% (ranging from 0.1% to 93.2%) were identified during this long-term observation period from January 2016 to March 2023 in southern Thailand. PP (93.2%), SL (33.8%), BT (29.1%), and ET (7.8%) had the highest infection rates over the 7-year study period (Table-1 and Figure-2).

**Table-1:** The overall prevalence of nine different gastrointestinal parasites identified in fighting bulls throughout the study period.

Parasites	No. of infection cases	Prevalence (%)	95% confidence intervals
Trematode			
*Paramphistome* spp.	3956	93.2	92.45–93.97
*Eurytrema* spp.	332	7.8	7.00–8.61
*Fasciola* spp.	187	4.4	3.79–5.03
*Moniezia* spp.	58	1.4	1.02–1.72
Nematodes			
Strongyles	1433	33.8	32.30–35.15
*Toxocara* spp.	17	0.4	0.21–0.59
*Trichuris* spp.	3	0.1	−0.01–0.15
Protozoa			
*Buxtonella* spp.	1236	29.1	27.76–30.50
*Eimeria* spp.	283	6.7	5.88–7.38

### Prevalence per year of GI parasite infection

A total of 3956 infected GI parasites were microscopically diagnosed during 2016–2023, with an average of approximately 521 cases per year. Significant variation in the prevalence of Helminth and protozoa infections, excluding TR -, across the 7 years of investigation (p < 0.05). Notably, PP consistently exhibited the highest prevalence among all GI parasites throughout the study period, reaching a peak annual prevalence of 97.9% in 2019. Meanwhile, the greatest prevalence of SL was 66.5% in 2023, 44.0% in 2023 for BT, 27% in 2016 for FL, 23.6% in 2016 for EM, 12.5% in 2016 for ET, and 0.4% in 2016 for MZ (Table-2).

**Table-2:** The prevalence of gastrointestinal parasite infections in fighting bulls in southern Thailand from January 2016 to March 2023, presented as positive numbers and percentages.

Year	No. of samples	Infected samples/year	Infected cases (No., %)								

			Helminth							Protozoa	
	
			PP	ET	FL	SL	TC	TR	MZ	BT	EM
2016	567	556 (98.1)	501 (88.4)	153 (12.5)	71 (27.0)	158 (27.9)	15 (2.6)	1 (0.2)	2 (0.4)	221 (39.0)	134 (23.6)
2017	595	578 (97.1)	557 (93.6)	80 (5.5)	33 (13.4)	133 (22.4)	2 (0.3)	1 (0.2)	2 (0.3)	76 (12.8)	95 (16.0)
2018	830	815 (98.2)	804 (96.9)	36 (3.0)	25 (4.3)	236 (28.4)	0 (0.0)	0 (0.0)	0 (0.0)	229 (27.6)	40 (4.8)
2019	958	951 (99.3)	938 (97.9)	8 (2.9)	28 (0.8)	294 (30.7)	0 (0.0)	0 (0.0)	11 (1.1)	229 (31.2)	1 (0.1)
2020	365	357 (97.8)	344 (94.2)	3 (1.4)	5 (0.8)	117 (32.1)	0 (0.0)	0 (0.0)	8 (2.2)	102 (27.9)	2 (0.5)
2021	160	158 (98.8)	150 (93.8)	2 (3.8)	6 (1.3)	57 (35.6)	0 (0.0)	0 (0.0)	13 (8.1)	48 (30.0)	1 (0.6)
2022	569	556 (97.7)	512 (90.0)	31 (5.4)	19 (3.3)	305 (53.6)	0 (0.0)	1 (0.2)	12 (2.1)	173 (30.4)	9 (1.6)
2023	200	200 (100.0)	150 (75.0)	19 (9.5)	0 (0.0)	133 (66.5)	0 (0.0)	0 (0.0)	10 (5.0)	88 (44.0)	1 (0.5)
Chi-square			187.191	118.366	433.693	254.951	84.129	4.365	98.852	128.250	483.334
p-value			0.000	0.000	0.000	0.000	0.000	0.737	0.000	0.000	0.000

PP=*Paramphistome* spp.; ET=*Eurytrema* spp.; FL=*Fasciola* spp.; SL=Strongyles; TC=*Toxocara* spp.; TR=*Trichuris* spp.; MZ=*Moniezia* spp.; BT=*Buxtonella* spp.; EM=*Eimeria* spp.

### Prevalence of GI parasite infection on different coastlines and seasons

Overall, the prevalence of GI parasitic infection was higher on the west coast (98.6%) than on the east coast (98.0%). A significant difference was observed in the prevalence of ET, SL, and BT infections between the east and west coasts (p < 0.05). SL and BT infection was more prevalent on the west coast, whereas ET infection was more prevalent on the east coast (Table-3).

**Table-3:** The prevalence of gastrointestinal parasite infections on different coastlines in southern Thailand during the study period, reported as positive numbers and percentages.

Coastlines	No. of samples	Infected samples	Infected cases (No., %)								

			Helminth							Protozoa	
	
			PP	ET	FL	SL	TC	TR	MZ	BT	EM
East coast	2382	2335 (98.0)	2235 (93.8)	113 (4.7)	78 (3.3)	823 (34.6)	0 (0.0)	1 (0.0)	36 (1.5)	596 (25.0)	76 (3.2)
West coast	901	888 (98.6)	845 (93.8)	22 (2.4)	26 (2.9)	345 (38.3)	0 (0.0)	1 (0.1)	18 (2.0)	266 (29.5)	24 (2.7)
Chi-square			0.002	8.764	0.318	4.054	-	0.512	0.962	7.004	0.609
p-value			0.963	0.003	0.573	0.044	-	0.474	0.327	0.008	0.435

PP=*Paramphistome* spp.; ET=*Eurytrema* spp.; FL=*Fasciola* spp.; SL=Strongyles; TC=*Toxocara* spp.; TR=*Trichuris* spp.; MZ=*Moniezia* spp.; BT=*Buxtonella* spp.; EM=*Eimeria* spp.

The prevalence of GI parasitic infections was higher during the rainy season (98.5%) than during the summer season (97.7%). The prevalence of FL and EM infections significantly increased during the summer compared with the rainy season on the east coast (p < 0.05). In contrast, the prevalence of ET and BT infections was notably higher in the rainy season than in the summer (p < 0.05). Conversely, the prevalence of SL and BT infections was significantly higher in the summer than in the rainy season (p < 0.05) on the west coast. However, the prevalence of FL infection was significantly higher during the rainy season than during the summer (p < 0.05) (Table-4).

**Table-4:** The prevalence of gastrointestinal parasite infections in different seasons along the east and west coastlines in southern Thailand throughout the study period, presented as positive numbers and percentages.

Seasons	No. of samples	Infected samples	Infected cases (No., %)								

			Helminth							Protozoa	
	
			PP	ET	FL	SL	TC	TR	MZ	BT	EM
East coast											
Summer	1209	1175 (97.2)	1143 (94.5)	37 (3.1)	58 (4.8)	407 (33.7)	(0.0)	0 (0.0)	20 (1.7)	262 (21.7)	48 (4.0)
Rainy	1172	1158 (98.8)	1091 (93.1)	76 (6.5)	20 (1.7)	417 (35.4)	(0.0)	1 (0.1)	16 (1.4)	334 (28.5)	27 (2.3)
Chi-square			2.167	15.434	17.943	0.801	-	1.032	0.334	14.776	5.418
p-value			0.141	0.000	0.000	0.371	-	0.310	0.563	0.000	0.020
West coast											
Summer	402	399 (99.2)	371 (92.3)	10 (2.5)	2 (0.5)	181 (45.0)	(0.0)	0 (0.0)	9 (2.2)	140 (34.8)	12 (3.0)
Rainy	499	488 (97.8)	474 (95.0)	12 (2.4)	24 (4.8)	162 (32.9)	(0.0)	1 (0.2)	9 (1.8)	126 (25.3)	12 (2.4)
Chi-square			2.782	0.006	14.772	13.930	-	0.807	0.215	9.969	0.289
p-value			0.095	0.936	0.000	0.000	-	0.369	0.643	0.002	0.591

PP=*Paramphistome* spp.; ET=*Eurytrema* spp.; FL=*Fasciola* spp.; SL=Strongyles; TC=*Toxocara* spp.; TR=*Trichuris* spp.; MZ=*Moniezia* spp.; BT=*Buxtonella* spp.; EM=*Eimeria* spp.

### Prevalence of GI parasite infection in different provinces

The overall results revealed that the highest prevalence of GI parasitic infections was observed in Chumphon (100%), followed by Trang (99.8%) and Phattalung (99.0%). PP, ET, SL, and BT infections varied significantly among the seven provinces in southern Thailand (p < 0.05) (Table-5). PP infection rates were highest in Trang (99.0%), followed by Phatthalung (98.5%) and Chumphon (97.6%). With regard to ET infection, the three provinces with the highest infection rates were Nakhon si Thammarat (5.5%), Surat Thani (4.7%), and Trang (3.5%). BT infection was dominant in Trang (31.9%), Surat Thani (28.5%), and Krabi (27.9%).

**Table-5:** The prevalence of gastrointestinal parasite infections in different provinces in southern Thailand throughout the study period, reported as positive numbers and percentages.

Provinces	No. of samples	Infected samples	Infected cases (No., %)								

			Helminth							Protozoa	
	
			PP	ET	FL	SL	TC	TR	MZ	BT	EM
Nakhon si Thammarat	1676	1649 (98.3)	1587 (94.8)	92 (5.5)	56 (3.3)	569 (33.9)	0 (0.0)	1 (0.1)	23 (1.4)	408 (24.3)	53 (3.2)
Suratthani	386	369 (95.6)	331 (85.8)	18 (4.7)	8 (2.1)	164 (42.5)	0 (0.0)	0 (0.0)	12 (3.1)	110 (28.5)	13 (3.4)
Phatthalung	201	199 (99.0)	198 (98.5)	1 (0.5)	7 (3.5)	58 (28.9)	0 (0.0)	0 (0.0)	1 (0.5)	53 (26.4)	5 (2.5)
Songkhla	80	79 (98.8)	79 (98.8)	1 (1.3)	3 (3.8)	22 (27.5)	0 (0.0)	0 (0.0)	0 (0.0)	19 (23.8)	3 (3.8)
Chumphon	41	41 (100)	40 (97.6)	1 (2.4)	4 (9.8)	10 (24.4)	0 (0.0)	0 (0.0)	0 (0.0)	6 (14.6)	2 (4.9)
Krabi	501	489 (97.6)	449 (89.6)	8 (1.6)	13 (2.6)	222 (44.3)	0 (0.0)	0 (0.0)	9 (1.8)	140 (27.9)	14 (2.8)
Trang	400	399 (99.8)	396 (99.0)	14 (3.5)	13 (3.3)	123 (30.8)	0 (0.0)	1 (0.3)	9 (2.3)	126 (31.5)	10 (2.5)
Chi-square			92.428	26.813	9.161	40.603	-	3.132	11.055	16.260	1.561
p-value			0.000	0.000	0.241	0.000	-	0.873	0.136	0.023	0.980

PP=*Paramphistome* spp.; ET=*Eurytrema* spp.; FL=*Fasciola* spp.; SL=Strongyles; TC=*Toxocara* spp.; TR=*Trichuris* spp.; MZ=*Moniezia* spp.; BT=*Buxtonella* spp.; EM=*Eimeria* spp.

### Prevalence of GI parasite infection among different age groups

In general, the prevalence of GI parasitic infections was highest (99.2%) in patients aged 7.0–7.9 years and 8.0–8.9 years and lowest (96.9%) in those aged 1.0–4.9 years (Table-6). The prevalence of PP infection differed significantly among the five age groups (p < 0.05), with the highest prevalence observed in the 9.0–9.9 age group (96.0%). However, there was no significant difference in the prevalence of other parasitic infections according to age group.

**Table-6:** The prevalence of gastrointestinal parasite infections for different age groups of fighting bulls throughout the study period, reported as positive numbers and percentages.

Age (years)	No. of samples	Infected samples	Infected cases (No., %)								

			Helminth							Protozoa	
	
			PP	ET	FL	SL	TC	TR	MZ	BT	EM
1.0–4.9	295	286 (96.9)	254 (86.1)	24 (8.1)	12 (4.1)	96 (32.5)	2 (0.7)	0 (0.0)	4 (1.4)	70 (23.7)	18 (6.1)
5.0–5.9	831	817 (98.3)	765 (92.1)	64 (7.7)	42 (5.1)	284 (34.2)	3 (0.4)	0 (0.0)	19 (2.3)	266 (32.0)	54 (6.5)
6.0–6.9	1063	1042 (98.0)	998 (93.9)	86 (8.1)	51 (4.8)	362 (34.1)	4 (0.4)	3 (0.3)	11 (1.0)	306 (28.8)	78 (7.3)
7.0–7.9	770	764 (99.2)	732 (95.1)	64 (8.3)	30 (3.9)	256 (33.2)	4 (0.5)	0 (0.0)	11 (1.4)	219 (28.4)	42 (5.5)
8.0–8.9	529	525 (99.2)	502 (94.9)	40 (7.6)	23 (4.3)	179 (33.8)	3 (0.6)	0 (0.0)	8 (1.5)	151 (28.5)	37 (7.0)
9.0–9.9	322	316 (98.1)	309 (96.0)	28 (8.7)	14 (4.3)	110 (34.2)	0 (0.0)	0 (0.0)	3 (0.9)	88 (27.3)	22 (6.8)
>9.9	426	414 (97.2)	388 (91.1)	26 (6.1)	14 (3.3)	143 (33.6)	1 (0.2)	0 (0.0)	2 (0.5)	135 (31.7)	31 (7.3)
Chi-square			39.484	2.550	3.038	0.407	2.843	8.944	9.184	9.952	3.112
p-value			0.000	0.863	0.804	0.999	0.828	0.177	0.164	0.127	0.795

PP=*Paramphistome* spp.; ET=*Eurytrema* spp.; FL=*Fasciola* spp.; SL=Strongyles; TC=*Toxocara* spp.; TR=*Trichuris* spp.; MZ=*Moniezia* spp.; BT=*Buxtonella* spp.; EM=*Eimeria* spp.

## Discussion

The overall prevalence of GI parasite infections in fighting bulls is 93.2%. Our findings agree with those of previous studies conducted on beef cattle across various regions of Thailand, including 61.0% in the northern province of Nan [10], 65.9% in the northeastern province of Kalasin [3], 86.4% in the western province of Kanchanaburi [11], and 87.3% in Nakhon Si Thammarat province in the southern region [12]. These results highlight that GI parasite infection remains a persistent challenge in cattle farming, especially in the southern region where infection rates are consistently high.

PP or rumen flukes, exhibited the highest prevalence (93.2%) among all GI parasites detected in this study. These findings are consistent with those of a recent study [9], which reported a high prevalence of PP infection in 2–5-year-old fighting bulls in southern Thailand (97.17%). However, our results demonstrate a higher prevalence of PP infection in beef cattle in northern Thailand (78.4% and 25.4%, respectively) compared to previous studies [13, 14]. PP are the most common trematodes found in the rumen and reticulum of ruminants. Evidence of its infection has been reported worldwide, especially in Asia (61.5%) [15]. Typically, mature rumen flukes do not induce clinical disease, but substantial infection with immature parasites can result in enteritis accompanied by symptoms such as diarrhea, anorexia, and dehydration, which may result in mortality among young livestock [16]. Due to the relatively high annual rainfall in southern Thailand and the presence of numerous river basins used for cattle farming, the region serves as a suitable environment for the growth of immature rumen fluke and increased freshwater snail population. These snails act as intermediate hosts for rumen flukes and contribute to a higher incidence of rumen flukes compared to other regions in Thailand. A suitable treatment and prevention strategy involves the administration of oxyclozanide, the only anthelmintic agent proven to be effective against rumen flukes [17, 18].

SL had the second highest infection rate and the annual prevalence increased significantly during the 7-year study period. Our findings indicate a higher prevalence of SL infection (33.7%) than that reported in previous studies [9, 13, 14]. The increase in the number of SL-positive bulls may indicate ineffective anthelmintic use, possibly due to parasitic resistance to the medication. Other contributing factors include insufficient healthcare practices and ineffective pasture management on farms.

ET and FL are zoonotic trematodes that can impact cattle productivity and raise concerns for public health [19, 20]. ET is a common trematode found in the pancreatic ducts of ruminants, typically infecting them through the ingestion of grasshoppers carrying metacercaria [21]. FL is a notable parasite located in the bile ducts of cattle, exerting a substantial negative effect on animal productivity [22]. The overall prevalence of ET and FL infection were 7.8% and 4.4%, respectively. Throughout the long-term study, the proportion of bulls tested positive for ET decreased from 12.5% in 2016 to a minimum of 1.4% in 2020. However, in the following years (2021–2023), the rate of infection resurgence. Moreover, ET infection had the highest incidence in Nakhon Si Thammarat province (5.5%). In contrast, the FL infection rate steadily declined from 27% in 2016 to 0% in 2023, with 9.8% of the dominant positive cases reported in Chumphon province. These findings reveal a higher incidence than that reported in a previous study, which reported an infection rate lower than 3.0% for both species [9]. These findings suggest that bulls infected with ET may act as carriers of the disease without exhibiting clinical signs, potentially spreading parasite eggs in grazing areas and surface water resources. Therefore, it is important to consider a regular deworming program with an effective anthelmintic agent and the elimination of intermediate hosts as important protocols for bull farmers to reduce the number of infections.

Throughout the study period, two species of protozoa with a high infection rate were identified: BT (29.1%) and EM (6.7%). Our findings are consistent with those of a previous survey of beef cattle in Nakhon Si Thammarat province [12], which reported a prevalence of BT infection of 16.36%. BT is a ciliated protozoon commonly found in the large intestine and results in subclinical and clinical colitis [23]. Cattle living in muddy environments with poor health conditions are highly susceptible to BT infection [24]. With regard to EM, the incidence of infection steadily declined from 23.6% in 2016 to 0.5% in 2023. EM infection occurs more frequently in young animals than in older animals [25]. Several factors, such as season, climate, and farm sanitation, can also influence the prevalence rate [26]. The fact that these bulls are mostly raised in individual stalls without grouping may contribute to a lower incidence of EM. However, further investigations are needed into the associated risks, such as the type of housing, bedding, floor, and hygiene conditions.

This study indicated that the prevalence of GI parasite infections is influenced by various factors, including geographical area, season, and age. These results show that there is a significant difference in the prevalence of GI parasite infection based on geographic area. In addition, endemic areas of GI parasite infection were found in all seven provinces of southern Thailand, accounting for more than 95% of the population. The prevalence of SL and BT was significantly higher on the west coast, whereas the prevalence of ET was significantly higher on the east coast. The western side is characterized by steep coasts, whereas the eastern side is characterized by river plains with large rivers and lakes, which serve as the main agricultural area in the region [27]. In addition, the western side experiences higher annual rainfall compared to the eastern side [28]. The diversity of landscapes and variations in annual rainfall in the southern region may contribute to different GI parasite infections. Our findings showed that the prevalence differed significantly according to season in Southern Thailand based on the geographic area. PP attained peak counts during both the summer and rainy seasons on both coastlines. Infection rates of some species (SL, ET, FL, BT, and EM) varied for different reasons depending on the geographic areas. In addition, PP infection prevalence was significantly higher in bulls aged 9.0–9.9 years than in other age groups. This age-related variation shows differences in exposure to infection, since older bulls may have a low immune response to defend against the infective stage of this parasite.

## Limitations

This study faced limitations due to its reliance on conventional diagnostic methods, which hindered the precise identification and quantification of GI parasitic infections. Additionally, it omitted factors such as management practices and nutritional status, which can influence infection rates in bulls. Future research should incorporate molecular diagnostic techniques and investigate the impact of management, nutrition, and genetics on susceptibility to parasitic infections to improve the health outcomes of fighting bulls.

## Conclusion

The prevalence of GI parasite infections remained consistently high over the 7 years from 2016 to 2023. PP infections were the most common, followed by SL and BT. The persistent increase in ET infection over the past several years warrants immediate attention, especially in view of the potential zoonotic implications. The prevalence of GI parasites differed between the western and eastern coasts of Southern Thailand for the species studied. Therefore, this study provided a comprehensive understanding of the distribution of GI parasitic infections within the study areas. This study will contribute to the development of basic preventive and control methods for the effective management of GI parasitic infections in bulls.

## Authors’ Contributions

DC: Research concept and drafted the manuscript. NC: Recorded data and performed GI parasite detection, identification, and imaging. KS: Designed the study and data analysis. WS: Collected and assembled data. WB: Designed the study, data analysis and interpretation, and revised the manuscript. All authors have read, reviewed, and approved the final manuscript.
